# TRAF7 contributes to tumor progression by promoting ubiquitin-proteasome mediated degradation of P53 in hepatocellular carcinoma

**DOI:** 10.1038/s41420-021-00749-w

**Published:** 2021-11-13

**Authors:** Qi Zhang, Xinqi Zhang, Weiguo Dong

**Affiliations:** Department of General Medicine, Renmin Hospital of Wuhan University, Wuhan, 430071 Hubei China

**Keywords:** Apoptosis, Cancer, Ubiquitylation

## Abstract

It has been proved that TRAFs family proteins played malfunctioning roles in the development of human cancers. TRAF7 is the last one of TRAFs family proteins to be found, which was demonstrated to be involved in a serious of cancers development. In this study, we systematically investigated the molecular mechanisms of TRAF7 in facilitating hepatocellular carcinoma (HCC). We discovered that TRAF7 was overexpressed in tumor tissues and the increased TRAF7 expression was closely associated with tumor size, histologic grade, TNM stage and poor prognostication. TRAF7 overexpression repressed cell apoptosis and promoted cell proliferation, invasion and migration, whereas knockdown of TRAF7 in HCC cells had totally opposite effects. Besides, we identified the interaction between TRAF7 and P53 in HCC and demonstrated that TRAF7 promoted ubiquitin-proteasome mediated degradation of P53 at K48 site. The rescue assays further proved that the function of TRAF7 in inhibiting apoptosis and promoting tumor development was depended on P53 in HCC. Overall, this work identified that TARF7 promoted tumorigenesis by targeted degradation P53 for ubiquitin-mediated proteasome pathway. Targeting the TRAF7-P53 axis may provide new insights in the pathogenesis of HCC, and pave the way for developing novel strategies for HCC prevention and treatment.

## Introduction

Hepatocellular carcinoma (HCC) is the fourth leading causes of cancer-related death and the most common type of primary hepatic neoplasm. More than 80 thousand patients were diagnosed with HCC and more than 70 thousand patients with HCC died during 2018 [1]. A variety of pathogenesis have been reported to be associated with HCC, among which Hepatitis B virus and Hepatitis C virus infection, alcohol-abusing, and non-alcohol fatty liver disease are the major factors for HCC occurrence [2]. But how HCC happens and its underling mechanisms remain poorly understood. Although plenty kinds of treatment methods such as hepatectomy and targeted drug therapy have been applied for HCC, the patients’ prognostication are still very poor [3]. Thus, there is an urgent need to identify new therapeutic targets and drugs for HCC treatment.

Tumor necrosis factor (TNF) receptor-associated factor (TRAF) family proteins were firstly reported as signal delivering components of TNF-R superfamily members [4]. TRAF family proteins share the same conserved molecular domain, including a RING finger domain and zinc finger domains [5]. Thus, TRAF family proteins function as E3 ubiquitin ligases and are involved in many molecular biological processes including survival, proliferation, differentiation, and autophagy [5–7]. TRAF7 was firstly identified as components of the NF-kB pathway and to induce its activation [8, 9]. As a member of the TRAF family, TRAF7 has been reported on regulating post-translational modification and innate immune responses [10, 11].

Previous studies have revolved that TRAF family members could function as both oncogene and tumor suppressor genes [12–14]. As the last member of the TRAF family to be discovered, the function of TRAF7 in the occurrence and development of several human cancers has been well studied [15]. For example, TRAF7 specifically interacted with MEKK3 and potentiated MEKK3-mediated AP1 and CHOP activation and induced caspase-dependent apoptosis [16], TRAF7 gene mutations drove meningioma tumorigenesis [17], what’s more, TRAF7 promoted ubiquitin-proteasome degradation of P53 in breast cancer [18]. However, the function and mechanisms of TRAF7 in HCC have not been fully studied, it’s pretty interesting to further understand the function of TRAF7 in HCC.

In this study, we detected the potential function of TRAF7 in HCC development and progression. TRAF7 was found to be associated with HCC malignant behavior and inhibit HCC cell apoptosis. Mechanically, TRAF7 served as an oncogene through facilitating ubiquitin-proteasome mediated degradation of P53 at the K48 site.

## Materials and methods

### Clinical specimens

In this study, all those patients were included, who had been diagnosed with HCC from 2017 to 2020, who underwent hepatectomy in Renmin hospital of Wuhan University of China, and those who did not receive any chemotherapy or radiation therapy before. Matched normal tissues were taken from liver tissue within 2 cm of the tumor margin during surgery. Fresh tissue samples were collected within 30 minutes after surgery and stored at liquid nitrogen until used. Paired tissue specimens were histologically confirmed by experienced pathologists. All patients included were informed and consented to the study. The use of human tumor tissues in this study complied with the ethical guidelines of the 1975 Declaration of Helsinki. In addition, the experimental plan and contents were sent to the Medical Ethics Committee of Renmin Hospital of Wuhan University before the project began, and the research was approved by the Committee.

### Cell culture

Human HCC cell lines Huh-7, SK-Hep1 and HCCLM3, human embryonic kidney 293 T cells were cultured in the Dulbecco’s modified Eagle’s medium (DMEM) supplemented with 10% FBS (F05-001-B160216; Bio-One Biotechnology, Guangzhou, China) and 1% penicillin-streptomycin (15140-122; Gibco, Carlsbad, CA, USA) in a 5% CO2 incubator. Cell lines were authenticated by STR profiling.

### Immunofluorescence analysis (IF)

Cells were co-transfected with Flag-TRAF7 and Ha-P53 plasmids were cultured and fixed with paraformaldehyde. After blocking with 8% goat serum albumin and permeabilization with 0.5% NP-40, the cells were sequentially incubated with the primary antibodies Flag and Ha and fluorescein-conjugated secondary antibody. Nuclei were stained with DAPI. Images were acquired with a confocal laser scanning microscope (TCS SP8; Leica, Wetzlar, Germany).

### Western blot analysis

RIPA lysis buffer were used to lyse tissues and cells, lysates were quantified with a BCA protein assay kit (23225, Thermo Fisher, Rockford, USA). Proteins were transferred to polyvinylidene fluoride (PVDF) after separation by 10% sodium dodecyl sulfate-polyacrylamide gel electrophoresis (SDS-PAGE). Subsequently, membranes were sequentially incubated with specific primary antibodies overnight and with HRP-labeled secondary antibody for another one hour. Finally, signals were detected with an ECL kit and visualized in a ChemiDoc MP Imaging System. Antibodies used in this study were provided in Supplementary Table 1.

### RNA extraction and Quantitative real-time PCR (qRT-PCR) analysis

Total RNA was extracted from tissues and cells using the Trizol reagent kit according to the manufacturer’s instructions. Reverse transcription was conducted by HiScript II QRT SuperMix (Vazyme Biotech Co., Ltd, Nanjing, China) to obtain cDNA. qRT-PCR assays were performed in a real-time PCR system (LightCycler 480 Instrument II, Roche Diagnostics Inc., Basel, BS, Switzerland), and mRNA expression was assessed by the comparative cycle threshold (Ct) method (2^−ΔCt^). The sequences of the primers used are listed in Supplementary Table 2.

### Cell transfections

Plasmids and the small interfering RNA (siRNA) targeting TRAF7 and P53 were obtained from GENECREATE (Wuhan, China), the transfection of cells were performed utilizing the GenMute (SignaGen, Maryland, USA) following the manufacturer’s protocol. The sequences of siRNA were present in Supplementary Table 3.

### Cell proliferation assays

CCK-8 and colony formation assays were performed to assess tumor cell proliferation abilities. For CCK-8, cells were cultured and then incubated with CCK-8 solution (Dojindo, Kumamoto Ken, Japan) for a duration of 2 h. Finally, absorbance was measured at 450 nm with the help of a microplate reader. Colony formation assay was performed by seeding 1000-2000 cells per well and were incubated for a period of two weeks. The desired clones were stabilized with 4% paraformaldehyde, stained with 0.1% crystal violet solution, and counted by microscope.

### Wound healing assay

HCC cells were starved without FBS after be sown in a 6-well plate for 24 h. a 100 ul plastic pipette tip was used to scratch cells on the bottom of the plates. Images of the wounds were captured at 0 and 24 or 48 h.

### Transwell assay

8 μl pore-size Matrigel-coated chambers were prepared and placed in 24-well transwell plates. Total 10% FBS DMEM was added in the lower chamber and HCC cells were sown in the upper chamber with serum-free medium. 42-72 h later, cells in the upper chamber were remover and the crossed cells were stabilized and dyed.

### Cell apoptosis analysis

Following the manufacture’s protocol of an Annexin V-fluorescein isothiocyanate (FITC) apoptosis detection kit (Beyotime, Shanghai, China), HCC cells were detected by FC500 flow cytometer (Beckman-Coulter) and apoptosis rate was counted.

### Co-Immunoprecipitation (Co-IP) and Ubiquitination assay

For endogenous co-IP, HCC cells transfected with Flag-tagged TRAF7 were lysed in immunoprecipitation buffer. Then cell lysates were incubated with Flag antibody or IgG and 10 μl of Protein A/G magnetic beads for 3 h. Subsequently, the beads were sequentially rinsed two times with high salt and low salt wash buffers. Western Blotting was followed with Flag and P53 primary antibodies. For Exogenous co-IP, HEK293T cells were co-transfected with Flag/Ha-tagged TRAF7 and P53, the next steps are the same as for endogenous co-IP protocols. For ubiquitination analysis, Huh-7 cells were co-transfected with the indicated plasmids were lysed in cold IP lysis buffer containing 1% SDS. Subsequently, the lysates were diluted tenfold with IP lysis buffer and subjected to ultrasonic pyrolysis and centrifugation. The next steps were the same as those used for co-IP.

### Statistical analysis

GraphPad Prism 8.0 software and SPSS 21.0 software were used for statistical analysis. Student’s *t* test or a nonparametric statistical analysis using the Mann-Whitney U test were performed to compare distinction between two groups and one-way ANOVA was performed for comparisons among multiple groups. TRAF7 expression levels were ranked and patients were divided into high and low groups based on the median. A low TRAF7 group was defined as a value lower than the 50^th^ percentile, while a high TRAF7 group was defined as a value greater than the 50th percentile, in each of 49 patients. Following the Kaplan-Meier methodology, the survival curves were plotted. All values are shown as the means ± SDs. *P* values were categorized as follows: *P < 0.05; **P < 0.01; **P < 0.01.

## Results

### TRAF7 was increased in HCC tumor tissues

**Fig. 1:**
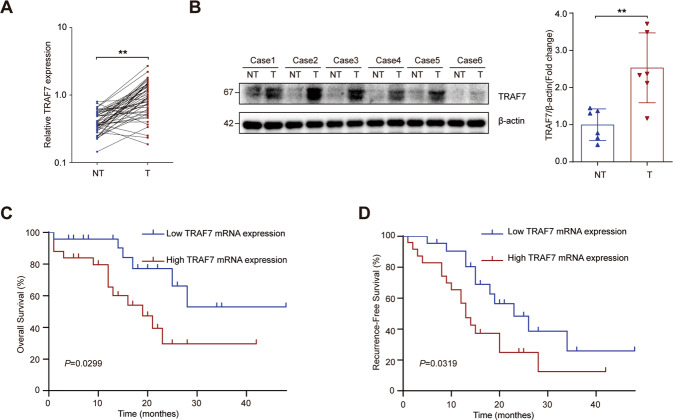
TRAF7 was upregulated in HCC. **A** TRAF7 mRNA expression level in HCC tissues and adjacent non-tumor tissues of 49 HCC patients. NT: Non-tumor tissue, T: Tumor tissue. *n* = 49. **B** Western Blot analysis of TRAF7 expression in adjacent non-tumor and HCC tissues. *n* = 6. **C**, **D** Kaplan-Meier analysis of overall survival (OS) and recurrence-free survival (RFS) based on TRAF7 expression levels in 49 patients with HCC. ^**^p < 0.01.

To investigate the role of TRAF7 in HCC, we firstly examined the TRAF7 expression profile by qRT-PCR and Western Blot assays. TRAF7 mRNA level was tested in 49 cases of patients with HCC between tumor and adjacent non-tumor tissues by qRT-PCR. The results indicated that the mRNA levels of TRAF7 in tumor tissues were upregulated compared with adjacent non-tumor tissues (Fig. 1A). What’s more, the Western Blot assay proved the increased TRAF7 protein level in tumor tissues (Fig. 1B). We then collected clinical characteristics of patients and the results were presented in Table 1, it was shown that TARF7 expression were significantly associated with tumor size, histologic grade, and TNM stage. Moreover, increased TARF7 expression tended to be associated with shorter overall survival (OS) and recurrence-free survival (RFS) (Fig. 1C, D) in HCC patients.

**Table 1:** Clinical characteristics of 49 HCC patients according to TRAF7 expression level.

Characteristics	Number of cases	TRAF7 expression		*p* Value
		Low(*n* = 25)	High(*n* = 24)	
*Age(years)*				0.290
≥60	36	20	16	
<60	13	5	8	
*Gender*				
Male	20	2	18	0.103
Female	29	23	6	
*Tumor size*				0.021*
≥5 cm	31	10	21	
<5 cm	18	15	3	
*Histologic grade*				
Poor or moderate	26	8	18	0.007**
Well	23	17	6	
*HBV infection*				0.762
Positive	37	18	19	
Negative	12	7	5	
*Liver cirrhosis*				0.257
Yes	32	14	18	
No	17	11	6	
*Serum AFP (μg/L)*				0.213
≥400	31	17	14	
<400	18	8	10	
*PVTT*				0.056
Yes	18	6	12	
No	31	19	12	
*TNM Stage*				0.015*
Stage III + IV	23	5	18	
Stage I + II	26	20	6	

Bold values indicates statistically significant *p < 0.05, **p < 0.01.

### Increased TRAF7 facilitated proliferation, invasion, migration and blocked apoptosis of HCC cells

**Fig. 2:**
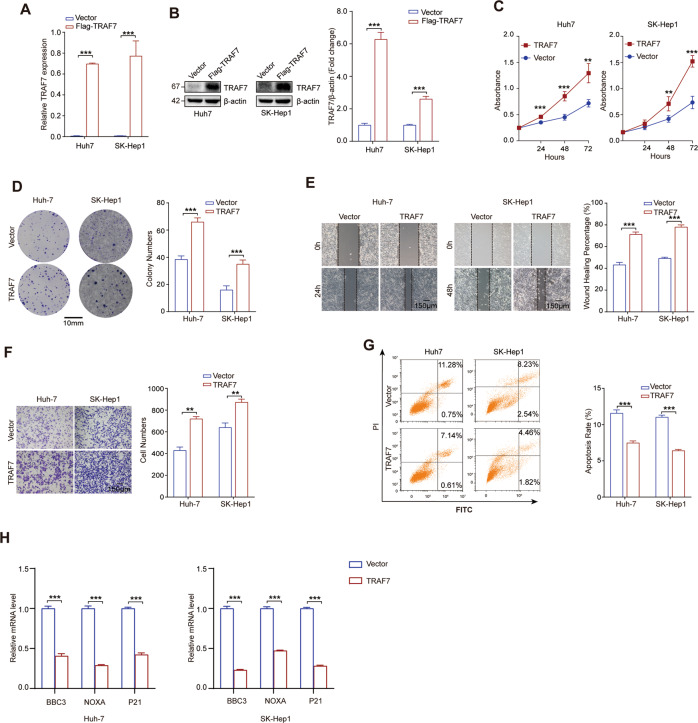
TRAF7 overexpression promoted hepatoma cells growth, proliferation, migration, invasion and repressed apoptosis. **A**, **B** qRT-PCR and Western Blot analysis identified TRAF7 plasmid transfection efficiency in Huh-7 and SK-Hep1 cells. **C**, **D** Cell Counting Kit-8 analyses and Colony formation indicated that TRAF7 facilitated hepatoma cells proliferation in Huh-7 and SK-Hep1 cells. **E**, **F** Wound-healing and transwell analyses showed enhanced migration and invasion abilities in TRAF7 overexpressed Huh-7 and SK-Hep1 cells. **G** Flow cytometry exhibited that TRAF7 inhibited cell apoptosis in Huh-7 and SK-Hep1 cell lines. **H** qRT-PCR detecting pro-apoptotic effector genes (BBC3, NOXA) and cell cycle arresting gene P21 in TRAF7 overexpressed Huh-7 and SK-Hep1 cells. All experiments were performed in triplicate. ^**^p < 0.01; ^***^p < 0.001.

Based on the potential role of TRAF7 in the process of HCC, we first sought to study the effects of TRAF7 on HCC cells malignant biological properties. TRAF7 plasmid transfection efficiency was detected by qRT-PCR and Western Blot in Huh7 and SK-Hep1 (Fig. 2A, B). TRAF7 overexpression significantly promoted HCC cells growth in Huh7 and SK-Hep1 cell lines according to CCK-8 assays (Fig. 2C), colony formation assays demonstrated that TRAF7 enhanced cell proliferation (Fig. 2D). The migration and invasion abilities of TRAF7-overexpressed HCC cell lines were detected by wound-healing and transwell assays, the results showed that TRAF7 overexpression reinforced HCC cells migration and invasion (Fig. 2E, F). Moreover, cell apoptosis rate was examined by flow cytometry and the results indicated that TRAF7 significantly reduced the apoptotic rate of Huh-7 and SK-Hep1 cells (Fig. 2G). qRT-PCR analysis indicated that TRAF7 overexpression significantly reduced pro-apoptotic effector genes BBC3, NOXA, and proliferation inhibition gene P21 expression (Fig. 2H).

### Knockdown of TRAF7 repressed proliferation, invasion, migration and induced apoptosis of HCC cells

**Fig. 3:**
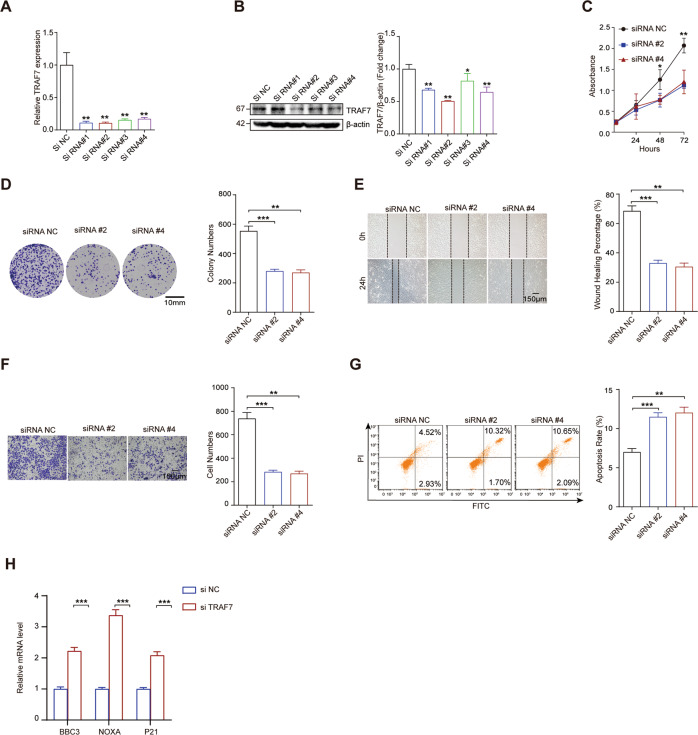
TRAF7 knockdown restrained hepatoma cells growth, proliferation, migration, invasion and induced apoptosis. **A**, **B** TRAF7 knockdown efficiency was identified by qRT-PCR and Western Blot assays. **C**, **D** CCK-8 and colony formation assays showed that TRAF7 knockdown inhibited HCCLM3 cells growth. **E**, **F** TRAF7 depletion restrained cell invasion and migration by transwell and wound-healing assays. **G** The knockdown of TRAF7 promoted cell apoptosis in HCCLM3. **H** qRT-PCR detecting the effect of TRAF7 knockdown in the expression of pro-apoptotic effector genes (BBC3, NOXA) and cell cycle arresting gene P21 in HCLM3 cells. All experiments were performed in triplicate. ^**^p < 0.01; ^***^p < 0.001.

We further examined the role of TRAF7 by decreasing its expression level in HCC cells. As shown in Fig. 3A, B, the 4 siRNA groups exhibited decreased TRAF7 mRNA and protein levels in the HCCLM3 cell line. As siRNA#2 and #4 produced the best reduction effects and were chosen for the next experiments. Functionally, growth curves detected by the CCK-8 assay showed that TRAF7 deletion inhibited HCC cell growth (Fig. 3C). Consistent with the CCK-8 results, colony numbers were obviously decreased in TRAF7 siRNA groups (Fig. 3D). Moreover, TRAF7 knockdown inhibited the migration and invasion of HCCLM3 cells compared with the NC group (Fig. 3E, F). Besides, flow cytometry results indicated that TRAF7 downregulation induced apoptosis of HCCLM3 cells (Fig. 3G). qRT-PCR analysis showed that TRAF7 down-regulation increased the expression of pro-apoptotic effector genes BBC3, NOXA, and proliferation inhibition gene P21 (Fig. 3H).

### TRAF7 promoted K48-linked ubiquitination and proteasome degradation of P53

**Fig. 4:**
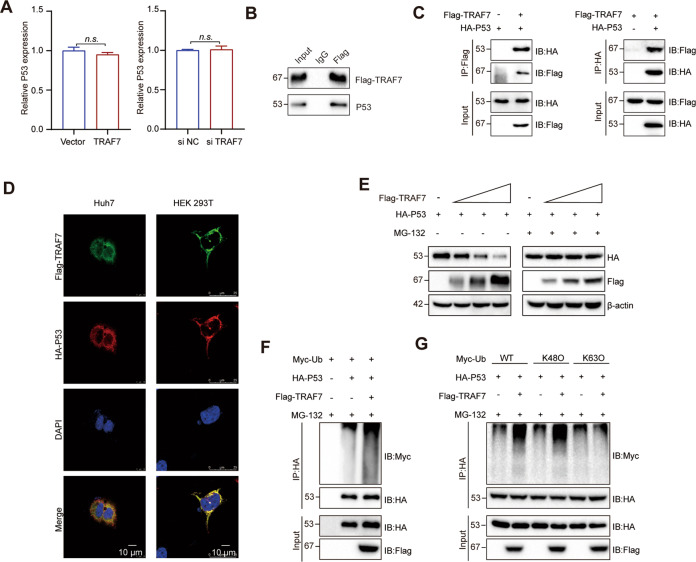
TRAF7 promoted K48-linked ubiquitination and degradation of P53. **A** the expression of P53 mRNA when TRAF7 modulated in HCC cells. **B** Endogenous co-IP assay showed that P53 was present in Flag immunocomplex, however absent in the IgG immunocomplex. **C** Exogenous co-IP assays were conducted to identify the interaction of TRAF7 and P53. **D** Immunofluorescence analysis showed TRAF7 and P53 co-located after co-transfected with Flag-TRAF7 and Ha-P53 in HEK-293T and Huh-7 cells. **E** P53 protein level after TRAF7 gradient transfected in Huh-7 cells with or without MG132. **F** Western blotting analysis of the ubiquitination of P53 in Huh-7 cells transfected with TRAF7 plasmid. MG132 was added for 6 h before harvest. **G** Ubiquitination screening of P53 by TRAF7 with indicated types of ubiquitin (K48O and K63O). ^**^p < 0.01; ^***^p < 0.001.

The TRAFs family is a kind of proteins in regulating complex signal pathways, and members of the family play diversified biological roles in a variety of diseases. Previous studies indicated that TRAF2, TRAF4, and TRAF6 could affect the stability of P53 protein and regulate tumor progress [19–21]. P53 is a key tumor suppressor and a crucial apoptosis pathway regulator in a variety of kinds of tumors [22]. Thus, the regulation relationship between the TRAF family and P53 may play a crucial role in tumorigenesis. What’s more, TRAF7 has been proved to affect the progression of breast cancer by regulating P53 stability [18]. Therefore, we were eager to explore whether TRAF7 plays the role in HCC through P53. Thus, we would like to detect the correlation of TARF7 and P53 of HCC. We firstly performed qRT-PCR to evaluate the P53 gene expression after perturbing TRAF7 levels. As shown in Fig. 4A, the mRNA levels of P53 were not altered among TRAF7 modulated groups and the control. Then endogenous co-IP assay was performed in Flag-tagged TRAF7 overexpressed Huh-7 cells, the results showed that P53 was conjugated with the Flag immunocomplex, while not with the IgG immunocomplex (Fig. 4B). Exogenous co-IP assays further confirmed that TRAF7 interacted with P53 in HEK 293 T cells (Fig. 4C). Moreover, the co-location of TRAF7 and P53 was present in Huh-7 and HEK 293 T cells by immunofluorescence analysis (Fig. 4D). It has been well established that TARF7 is an E3 ubiquitin ligase and promotes the ubiquitination of proteins [18]. We therefore speculated that TRAF7 regulates the stabilization of P53 via ubiquitination. Western blotting assays indicated that Ha-tagged P53 protein was significantly decreased after gradient transfection with Flag-tagged TRAF7 in Huh-7 cells. However, the addition of a 26 S proteasome inhibitor (MG132), completely abolished TRAF7-induced degradation of P53 (Fig. 4E), indicating that TRAF7 facilitated the proteasomal degradation of P53. Consistent with this finding, the ubiquitination of P53 was significantly increased by TRAF7 overexpression (Fig. 4F). K63 and K48 are the most common ubiquitination sites and different types of ubiquitination lead to distinct molecular processes. Subsequently, the ubiquitination assay results revealed that TRAF7 specifically promoted the addition of K48-linked polyubiquitin chains to P53 (Fig. 4G).

### The function of TRAF7 in HCC depends on P53

**Fig. 5:**
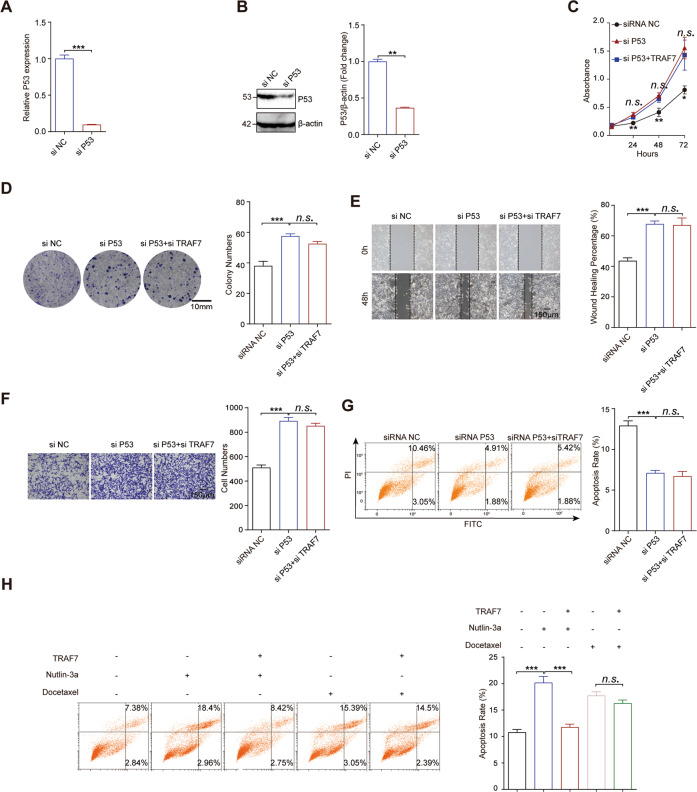
The function of TRAF7 in HCC cells depends on P53. **A**, **B** P53 knockdown efficiency was identified by qRT-PCR and Western Blot assays in SK-Hep1 cells. **C**, **D** CCK-8 and colony formation assays of cells transfected with both siP53 and siTRAF7. **E**, **F** P53 knockdown blocked siTRAF7 induced cells migration and invasion changes. **G** TRAF7 depletion failed to induce cell apoptosis in P53 knockdown SK-Hep1. **H** The effect of TRAF7 overexpression on nutlin-3a and docetaxel induced apoptosis. All experiments were performed in triplicate. ^**^p < 0.01; ^***^p < 0.001.

To further investigate whether TRAF7 regulated HCC cell malignant behaviors through P53, experiments were performed in P53 knockdown HCC cells. Firstly, P53 knockdown efficiency was confirmed by qRT-PCR and Western Blot (Fig. 5A, B). Notably, CCK-8 and colony formation assays showed that P53 deficiency abrogated the cells growth and proliferation changes induced by TRAF7 depletion (Fig. 5C, D). Besides, wound healing and transwell assays indicated that TRAF7 overexpression could not affect the migration and invasion abilities of P53 knockdown HCC cells (Fig. 5E, F). Moreover, consistent with the robust change in cell growth, migration and invasion abilities, the decreased cell apoptosis rate in TRAF7 overexpressed cells were blocked by P53 depletion (Fig. 5G). Thus, we proposed the potential mechanism of the TRAF7-P53 axis in the pathogenesis of HCC. To further confirm the crucial function of P53 in TRAF7 modulating apoptosis pathways, we applied P53 depended on apoptosis inducer nutlin-3a and P53-independent apoptosis inducer docetaxel. The results indicated that TRAF7 overexpression almost abolished nutlin-3a induced HCC cells apoptosis but made no difference to docetaxel-induced apoptosis (Fig. 5H).

Collectively, these data indicated that P53 was required for the function of TRAF7 in the pathogenesis of HCC.

## Discussion

HCC is becoming a global challenge because of its high morbidity and mortality, thus it’s urgent to establish efficient diagnostic and therapeutic strategies to improve the overall survival [23]. Here, we detected the increased expression of TRAF7 in tumor compared with non-tumor tissues, and found the relation between TRAF7 expression with clinical characteristics and prognostication. The results indicated that TRAF7 was markedly associated with tumor size, histologic grade, and TNM stage. Subsequently, cancer malignant behavior assays were performed and the results demonstrated that TRAF7 exerted predominant oncogene effects on tumor cells proliferation, migration, invasion in vitro. What’s more, TRAF7 significantly reduced the cells apoptosis rate of HCC. Therefore, we reasoned that the effects of TRAF7 on HCC progress might be leaded by inhibiting cell apoptosis.

Apoptosis is one of the major mechanisms in regulating cell proliferation and death balance [24]. Apoptosis disruption is recognized to be a leading cause of uncontrolled cell growth and tumorigenesis [25]. The decrease of programmed cell death is associated with abnormal cell proliferation and even malignant behavior [26]. P53 is one of the most famous downregulated genes in cancer and has been extensively studied. The predominant mechanism of P53 in cancer is its effects on the cell cycle and apoptosis [27]. Targeting P53 is raising to be an efficient strategy for cancer therapy. Ubiquitination by E3 ligases is capable to induce the relocation of p53 in tumor cells and determine the outcome of p53-mediated cell proliferation, apoptosis, and efficacy of cancer therapy [28]. Thus, we turned to study the relationship of TRAF7 and P53 in HCC.

Previous studies indicated that all 7 TRAF family members were altered in human cancers, with a gain of function usually for TRAF1 1, 4, 5, and 6, and loss of function usually for TRAFs 3 and 5 [29]. It has been also well proved that TRAF proteins play important roles in cancer. Interestingly, also TRAF3 is frequently decreased in human cancers, studies showed that TRAF3 can both positively and negatively regulate tumorigenesis [30]. It is evident from the previous studies that TRAF7 served as a tumor regulator in various cancers. The deletion of TRAF7 was found in 67% of malignant mesothelioma patients’ malignant cells in pleural fluids. TRAF7 mutation predominantly contributed to improving the diagnosis, prognosis, and therapy of patients with meningiomas [15, 31, 32]. However, how TRAF7 regulates HCC via P53 is still largely unknown. The present study demonstrated that TRAF7 binds to P53 and promotes its ubiquitination degradation at the K48 site, subsequently aggravates HCC progress.

It has been proved that P53 is primarily regulated by MDM2 through 3 major mechanisms: MDM2 directly ubiquitinates P53 by its E3 ligase activity and inducing P53 proteasomal degradation; MDM2 blocks P53 from binding with target DNA; MDM2 promotes export of P53 out of the cell nucleus. Blocking the interaction of MDM2 and P53 provides effective treatments for human cancer by activating the function of P53 in inhibiting tumor function. Several inhibitors have been designed and are already in clinical trials for cancer treatment [33]. Although MDM2 is crucial in regulating P53, it’s not likely that only MDM2 is responsible in P53 regulation. For example, Pirh2 encoded protein appears to have ubiquitin-protein ligase activity, which can promote ubiquitination and degradation of P53 independently of MDM2 [34]. Our study demonstrated that TRAF7 binds to P53 and promotes its ubiquitination degradation, which indicated that TRAF7 have an effect on P53 independently of MDM2. From this perspective, TRAF7 and MDM2 play a synergistic role in the regulation of P53. Besides, considering that both of them are E3 ubiquitin ligases, it is also very interesting to study the possible interaction and ubiquitination modification relationship between them. However, due to the limitations of this study, the interaction between TRAF7 and MDM2 was not further analyzed and this is where we’re going to go next.

In conclusion, the study demonstrated the hypothesis that TRAF7 positively regulated tumorigenesis, which promoted cell growth, migration, invasion, and blocked cells apoptosis. Besides, we identified P53 as the downstream target of TRAF7 and ubiquitin-proteasome-mediated degradated in HCC induced by TRAF7. The finding provided a promising tumorigenesis mechanism that might be helpful to HCC treatment.

## Supplementary information

supplementary tables

## Data Availability

The data that support the findings of this study are available from the corresponding author upon reasonable request.
