# Supplementary material for: TRAF7 contributes to tumor progression by promoting ubiquitin-proteasome mediated degradation of P53 in hepatocellular carcinoma
Source: Cell Death Discov. 2021 Nov 13;7:352. doi: 10.1038/s41420-021-00749-w (PMC8590685; doi:10.1038/s41420-021-00749-w)
Supplement: Supplementary file 1 — supplementary tables [file 41420_2021_749_MOESM1_ESM.docx]

**Supplementary Table S1. Sequences of primers used for PCR in this study**

| Primer names | sequences |
| --- | --- |
| β-actin forward | 5’-CATGTACGTTGCTATCCAGGC-3’ |
| β-actin reverse | 5’-CATGTACGTTGCTATCCAGGC-3’ |
| TRAF7 forward | 5’-AGGCTCCTACGACCCTCAG-3’ |
| TRAF7 reverse | 5’-AGCCGCTGTAGAGTTTGCAC-3’ |
| P53 forward | 5’-CAGATCCTAGCGTCGAGCCCC-3’ |
| P53 reverse | 5’-CTGGGTCTTCAGTGAACCATTGTTC-3’ |
| BBC3 forward | 5’-CTGATGGACTCAGCATCGGA-3’ |
| BBC3 reverse | 5’-CAGCACAACAGCCTTTCCTG-3’ |
| NOXA forward | 5’-TGGAAGTCGAGTGTGCTACTCAAC-3’ |
| NOXA reverse | 5’-CAGAAGAGTTTGGATATCAGATTCAGA-3’ |
| P21 forward | 5’-TGGAGACTCTCAGGGTCGAAA-3’ |
| P21 reverse | 5’-TTCCTCTTGGAGAAGATCAGCC-3’ |

**Supplementary Table S2. Sequences of siRNAs in this study**

| Names | sequences |
| --- | --- |
| siRNA NC | 5’-UUCUCCGAACGUGUCACGUTT-3’ |
| SiRNA-TRAF7 #1 | 5’-CGGGACGCAUCCAUGUUAAAUTT-3’ |
| siRNA -TRAF7 #2 | 5’-CUACUCCAUUGCUGUGACAAATT-3’ |
| siRNA-TRAF7 #3 | 5’-GACCAGAAUGGAAACGACCUUTT-3’ |
| siRNA-TRAF7 #4 | 5’-GCACUGUGAAGGUUUGGACUUTT-3’ |
| siRNA -P53 | 5’-GACTCCAGTGGTAATCTAC-3’ |

**Supplementary Table S3. Primary antibodies used in this study**

| Antigens | Manufacturer | Catalog Number | Application |
| --- | --- | --- | --- |
| TRAF7 | Abclonal | A3095 | 1:1000 for WB |
| β-Actin | Abclonal | AC026 | 1:10000 for WB |
| FLAG-tag | MBL | M185-3LL | 1:10000 for WB; 1:200 for IF; 1:500 for IP |
| HA-tag | Cell signaling technology | #3724 | 1:1000 for WB; 1:200 for IF; 1:500 for IP |
| MYC-tag | MBL | M047-3 | 1:1000 for WB |
| Goat anti-mouse IgG-HRP | Jackson | #115-035-003 | 1:5000 for WB |
| Goat anti-rabbit IgG-HRP | Jackson | #111-035-003 | 1:5000 for WB |
| Alexa Flour 568 goat anti-Rabbit IgG | Invitrogen | #A11036 | 1:200 for IF |
| Alexa Flour 488 goat anti-mouse IgG | Invitrogen | Cat#A11029 | 1:200 for IF |
